# The therapeutic window: timing dentoalveolar surgery to minimize MRONJ risk in denosumab-treated osteoporotic patients

**DOI:** 10.1093/jbmrpl/ziag038

**Published:** 2026-03-12

**Authors:** Daya Masri, Eli Rosenfeld, Shaked Adut, Gavriel Chaushu, Gloria Tsvetov, Gal Avishai, Omar Ghanaiem, Ehud Jonas, Ayelet Zlotogorski-Hurvitz, Daniel Muchnik

**Affiliations:** Department of Oral and Maxillofacial Surgery, Rabin Medical Center—Beilinson Hospital, Petach Tikva 49414, Israel; Department of Oral and Maxillofacial Surgery, The Maurice and Gabriela Goldschleger School of Dental Medicine, Tel Aviv University, Tel Aviv 69978, Israel; Department of Oral and Maxillofacial Surgery, Rabin Medical Center—Beilinson Hospital, Petach Tikva 49414, Israel; Department of Oral and Maxillofacial Surgery, The Maurice and Gabriela Goldschleger School of Dental Medicine, Tel Aviv University, Tel Aviv 69978, Israel; Department of Oral and Maxillofacial Surgery, Rabin Medical Center—Beilinson Hospital, Petach Tikva 49414, Israel; Department of Oral and Maxillofacial Surgery, The Maurice and Gabriela Goldschleger School of Dental Medicine, Tel Aviv University, Tel Aviv 69978, Israel; Department of Oral and Maxillofacial Surgery, Rabin Medical Center—Beilinson Hospital, Petach Tikva 49414, Israel; Department of Oral and Maxillofacial Surgery, The Maurice and Gabriela Goldschleger School of Dental Medicine, Tel Aviv University, Tel Aviv 69978, Israel; Department of Endocrinology, Diabetes and Metabolism, Rabin Medical Center—Beilinson Hospital, Petach Tikva 49414, Israel; Department of Oral and Maxillofacial Surgery, Rabin Medical Center—Beilinson Hospital, Petach Tikva 49414, Israel; Department of Oral and Maxillofacial Surgery, The Maurice and Gabriela Goldschleger School of Dental Medicine, Tel Aviv University, Tel Aviv 69978, Israel; Department of Oral and Maxillofacial Surgery, Rabin Medical Center—Beilinson Hospital, Petach Tikva 49414, Israel; Department of Oral and Maxillofacial Surgery, Rabin Medical Center—Beilinson Hospital, Petach Tikva 49414, Israel; Oral Medicine Unit, Department of Otolaryngology Head and Neck Surgery and Maxillofacial Surgery, Tel-Aviv Sourasky Medical Center, Tel Aviv 69978, Israel; Department of Oral Pathology, Oral Medicine and Maxillofacial Imaging, The Maurice and Gabriela Goldschleger School of Dental Medicine, Tel Aviv University, Tel Aviv 69978, Israel; Department of Oral and Maxillofacial Surgery, Rabin Medical Center—Beilinson Hospital, Petach Tikva 49414, Israel

**Keywords:** denosumab, osteoporosis, dentoalveolar intervention, MRONJ, osteonecrosis of the jaw, rebound effect, timing

## Abstract

Osteoporosis patients treated with denosumab (Dmab) remain at risk for medication-related osteonecrosis of the jaw (MRONJ), particularly after dentoalveolar surgery. Optimizing surgical timing may reduce this risk while avoiding complications related to prolonged drug interruption. This retrospective cohort study evaluated 258 osteoporotic patients receiving Dmab and examined both patient-level data and intervention-level data from 91 patients who underwent 185 dentoalveolar procedures. Multivariable logistic regression and generalized estimating equations (GEE) were applied to identify factors associated with MRONJ development. Medication-related osteonecrosis of the jaw occurred in 5.43% of the total cohort. All MRONJ cases at the intervention level were observed when dentoalveolar procedures were performed within 3 mo of a Dmab injection. A longer interval between the last Dmab dose and dentoalveolar intervention demonstrated a strong protective effect against MRONJ (OR 0.35, *p* = .0089). In addition, prolonged previous antiresorptive drug intake was identified as a significant independent risk factor at the patient level. No specific type of dentoalveolar procedure showed a statistically significant association with MRONJ development. These findings confirm that surgical timing is a critical determinant of MRONJ risk in Dmab-treated osteoporotic patients. Avoiding dentoalveolar interventions during the early post-injection period, particularly within the first 3 mo, may substantially reduce risk. A delay of at least 4 mo after the last Dmab administration appears to be a clinically important preventative strategy, balancing MRONJ avoidance with the need to minimize fracture risk resulting from prolonged discontinuation of therapy. Additional prospective studies are warranted to refine surgical timing recommendations and guide individualized patient care.

## Graphical Abstract

**Graphical Abstract:**
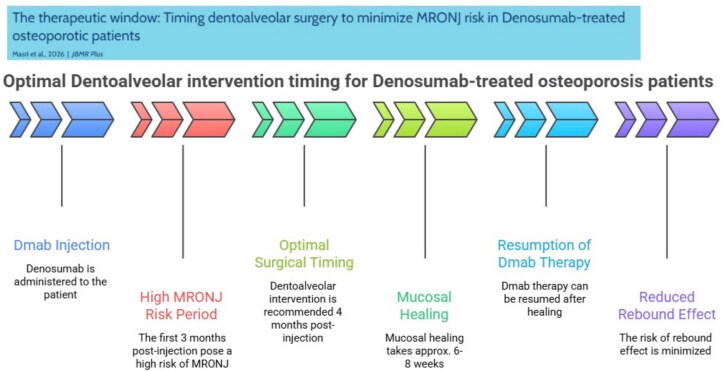

## Introduction

Osteoporosis, a prevalent skeletal disorder affecting approximately 10 million individuals in the United States and 18.3% of the global population is a major public health issue.^1^^,^^2^ It is characterized by diminished bone strength and an elevated susceptibility to fractures.^1^^,^^3^ Both bisphosphonates (BPs) and denosumab (Dmab), a human receptor activator of nuclear factor kappa-B ligand (RANKL) monoclonal antibody, are commonly employed antiresorptive drugs (ARD) to manage osteoporosis and mitigate fracture risk.^4^^,^^5^ However, the use of these agents has been associated with medication-related osteonecrosis of the jaw (MRONJ), a rare but debilitating condition defined as exposed bone, or bone that can be probed through an intraoral or extraoral fistula, in the maxillofacial region that has persisted for more than 8 wk in patients with a history of treatment with antiresorptive or anti-angiogenic drugs, and where there has been no history of radiation therapy to the jaw or no obvious metastatic disease to the jaws.^6–8^

While the precise pathogenesis of MRONJ remains incompletely understood, it is believed to involve a complex interplay of factors, including suppressed bone turnover, impaired angiogenesis and local infection or inflammation.^9–12^ Invasive dental procedures, particularly tooth extractions, ill-fitting dentures, and existing inflammatory dental diseases (eg, periodontal disease) have been frequently implicated as precipitating events in MRONJ development.^6^^,^^13–15^

Dmab, a monoclonal antibody targeting RANKL, reduces fracture risk through a mechanism different from BPs but is also associated with MRONJ, though its risk profile may differ. Given its 28-d half-life, it is believed that Dmab will not be retained in the body for long periods, and short-term follow-ups have demonstrated its low likelihood of inducing MRONJ. However, some studies indicate Dmab may pose similar or greater MRONJ risk than BPs.^6^^,^^16^ Emerging evidence indicates a potentially lower long-term risk of MRONJ with Dmab in osteoporotic patients, particularly after 2 yr of use.^17^ Campisi et al. have proposed a “pragmatic window of opportunity” to reduce MRONJ risk in patients treated with Dmab.^18^ They suggested that dental surgery, such as tooth extractions, may be safely performed within a specific timeframe (between 5 and 7 mo) following Dmab administration, when bone turnover starts to recover. However, this approach presents a clinical dilemma. Postponing the next Dmab injection to align with this window could increase the risk of fractures due to the “rebound effect”—a rapid decrease in bone density that occurs as the drug’s effectiveness diminishes.^7^^,^^19^^,^^20^ This may lead to an increased occurrence of multiple vertebral fractures according to the available evidence.^20^^,^^21^ Careful patient selection, interdisciplinary communication, strategic timing of Dmab injections, and meticulous postoperative care are crucial components of this proposed approach.

To the best of our knowledge, there are no studies that have examined the optimal time for performing dental treatments in patients treated with Dmab. The aim of this study is to evaluate the best timing of dentoalveolar interventions in osteoporotic patients on Dmab, with the aim of preventing MRONJ and avoiding the “rebound effect” and spontaneous vertebral fractures associated with Dmab discontinuation.

## Materials and methods

### Study cohort

**Figure 1:**
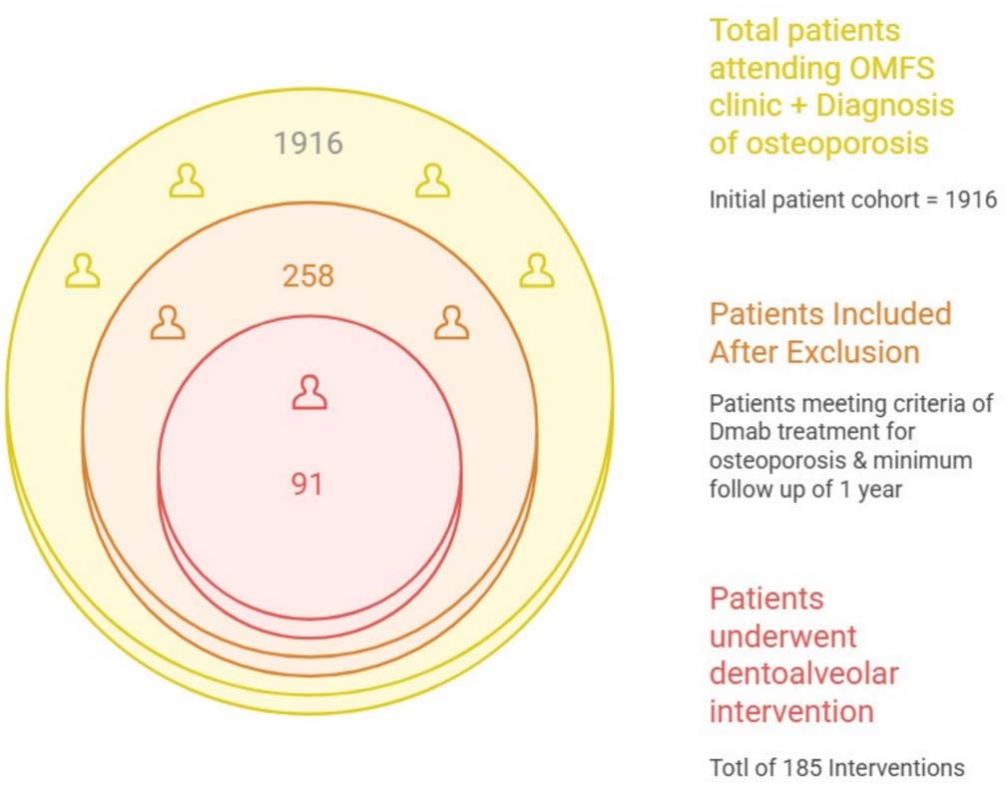
Inclusion process of the study cohort.

A retrospective cohort study including all patients who met the inclusion criteria and attended or referred to the Department of Oral and Maxillofacial Surgery (OMS) at a tertiary Medical Center (Rabin Medical Center, Campus Beilinson, Petah-Tikva, Israel), between the years 2013-2023. Data were extrapolated from hospital’s medical records by cross—referencing osteoporotic patients visited in the outpatient Oral and Maxillofacial Surgery Clinic (Figure 1).

### Inclusion criteria

(1) Patients diagnosed with osteoporosis, who visited the OMS outpatient clinic between the years 2013-2023 and were on ongoing treatment of Dmab for treatment of osteoporosis.
(2) Patients with at least 1 yr of follow-up.

### Exclusion criteria

(1) Patients who received Dmab for oncology indications.
(2) Patients with active anti-cancer treatment (chemotherapy/immunotherapy treatment).
(3) Patients who were irradiated to the maxillofacial region.

### Data collection

Data were collected from hospital’s medical records via “Research authority,” using a structured form, and with patient anonymization.

Demographic and medical history data included:

(1) Age.
(2) Gender.
(3) Relevant medical comorbidities according to the hospital medical records, which might affect occurrence of MRONJ—hypothyroidism, diabetes mellitus (DM), asthma/COPD, autoimmune disease, smoking, hypertension (HT), ischemic heart disease, hyperlipidemia, and history of neoplasm.
(4) History of corticosteroids administration.
(5) History of other ARDs prior to Dmab administration, including type and duration of treatment.

Surgical procedure and MRONJ related data included:

(1) Whether any surgical intervention involving bone (tooth, implant extraction, bone augmentation, and dental implant exposure) has been made.
(2) Timing of dentoalveolar intervention from last Dmab injection.
(3) Number of Dmab injections prior to dentoalveolar intervention.
(4) Previous treatment and duration of treatment with BPs prior to dentoalveolar intervention.
(5) Timing of first injection after the dentoalveolar intervention.
(6) Location of dentoalveolar intervention and MRONJ (maxilla/mandible).
(7) MRONJ development (Yes/No)—According to AAOMS 2022 definition.
(8) Dental implants presence around MRONJ sites.

MRONJ stage according to AAOMS 2022 definition.^6^

Our data structure included 2 levels: “patient level,” capturing the characteristics of the full cohort of patients seen in our clinic, and “intervention level,” focusing on the subset of patients who underwent surgical procedures. Given that patients could undergo more than one intervention, individual patients could contribute to multiple data points at the intervention level.

### Statistical analysis

All data were analyzed using Python version 3.12, with the Pandas library (version 2.1.3) for data manipulation, and Statsmodels (version 0.14.1) for statistical computations. For descriptive statistics, means and SDs were calculated for numeric variables. The Kolmogorov–Smirnov test was employed to assess the normality of the distribution for numeric variables. For categorical variables, frequencies and percentages were calculated.

At the intervention level, generalized estimating equations (GEE) were utilized to account for within-subject correlation due to multiple interventions per patient and potential clustering effects. The GEE models were adjusted for subject clustering, assumed a binomial distribution with a logit link function, and employed an exchangeable working correlation structure.

At the patient level, Chi-squared tests were performed for categorical variables and Kruskal–Wallis tests for numeric variables. To assess and mitigate multicollinearity, a variance inflation factor (VIF) test was conducted, with variables exhibiting a VIF greater than 5 being considered for exclusion or further investigation.

Subsequently, for variables with a *p*-value <.1 in the univariable analysis and no evidence of multicollinearity, a multivariable analysis was performed to assess potential interactions. Generalized estimating equations was used for multivariable analysis at the intervention level, while logistic regression was employed at the patient level. A *p*-value <.05 was considered statistically significant for all analyses.

## Results

Our study included 258 patients who met the inclusion criteria (Table 1), with an average age of 71.35 ± 8.26 yr, and predominantly females (91.47%). The mean number of Dmab injections was 6.33 ± 5.05, and the average duration of ARD intake was 9.16 ± 5.46 yr. Key comorbidities observed included HT (44.57%), hyperlipidemia (35.66%), and DM (20.54%). Overall, MRONJ was observed in 5.43% (14/258) of the total patient cohort.

**Table 1:** Total cohort characteristics (patient level).

**Characteristic**	**Subgroup**	***N***	**%**	**M**	**SD**
**Total**		258			
**Gender**	Male	22	8.53		
	Female	236	91.47		
**Tobacco smoking**	Yes	8	3.10		
**Autoimmune disease**	Yes	21	8.14		
**Hypertension**	Yes	115	44.57		
**Hypothyroidism**	Yes	59	22.87		
**Diabetes mellitus**	Yes	53	20.54		
**Asthma or COPD**	Yes	29	11.24		
**Ischemic heart disease**	Yes	33	12.79		
**Hyperlipidemia**	Yes	92	35.66		
**Intervention**	Yes	91	35.27		
**MRONJ**	Yes	14	5.43		
**Age (yr)**				71.35	8.26
**Number of injections**				6.33	5.05
**Duration of ARD intake (yr)**				9.16	5.46

### Patient-level analysis

Univariate analysis at the patient level (Table 2) demonstrated that the duration of ARD intake was significantly associated with MRONJ development (*p* = .005). Multivariable logistic regression analysis at the patient level (Table 3) confirmed this finding, with the duration of ARD intake remaining a significant independent risk factor (OR 1.25, 95% CI: 1.10-1.41, *p* < .001). Although age showed a trend toward an association with MRONJ (OR 1.09, 95% CI: 0.99-1.20, *p* = .067), it did not reach statistical significance in this model. Notably, the number of Dmab injections was not a significant independent predictor of MRONJ development at either the patient or intervention level. Ninety-one patients underwent 185 dentoalveolar interventions (Table 4). At the intervention level, univariate logistic regression analysis identified several significant associations with MRONJ development (Table 5). Implant presence at MRONJ site, was associated with an increased risk (OR 2.45, 95% CI: 1.02-5.86, *p* = .044). Both the duration of ARD intake (OR 1.19, 95% CI: 1.05-1.36, *p* = .0077) and age (OR 1.07, 95% CI: 1.00-1.14, *p* = .0525) also showed significant or borderline significant associations. Importantly, dentoalveolar intervention delay exhibited a strong protective association with MRONJ (OR 0.26, 95% CI: 0.12-0.57, *p* = .0008), suggesting that a longer delay between the last Dmab dose and intervention was associated with a reduced risk of MRONJ.

**Table 2:** Patient level univariate comparison regarding MRONJ development.

**Variable**	**Subgroup**	**MRONJ (*n* = 14)**	**%**	**No-MRONJ (*n* = 244)**	**%**	***p*-value**
**Gender**	Male	1	7.14	21	8.61	1
	Female	13	92.86	223	91.39	
**Tobacco smoking**	Yes	1	7.14	7	2.87	.9168
	No	13	92.86	237	97.13	
**Autoimmune disease**	Yes	1	7.14	20	8.20	1
	No	13	92.86	224	91.80	
**Hypertension**	Yes	6	42.86	109	44.67	1
	No	8	57.14	135	55.33	
**Hypothyroidism**	Yes	6	42.86	53	21.72	.13
	No	8	57.14	191	78.28	
**Diabetes mellitus**	Yes	2	14.29	51	20.90	.7981
	No	12	85.71	193	79.10	
**Asthma or COPD**	Yes	2	14.29	27	11.07	1
	No	12	85.71	217	88.93	
**Ischemic heart disease**	Yes	1	7.14	32	13.11	.81
	No	13	92.86	212	86.89	
**Hyperlipidemia**	Yes	6	42.86	86	35.25	.77
	No	8	57.14	158	64.75	
**Intervention**	Yes	14	100.00	77	31.56	0
	No	0	0.00	167	68.44	
**Age (yr)**		76.36 ± 7.56		71.06 ± 8.22		.01
**Number of injections**		8.07 ± 7.24		6.22 ± 4.88		.55
**Duration of ARD intake (yr)**		14.58 ± 7.01		8.88 ± 5.24		.005

**Table 3:** Multivariable logistic regression analysis for MRONJ risk factors (patient level).

**Variable**	**OR**	**95% CI**	***p*-value**
**Age (yr)**	1.09	0.99-1.20	.067
**Duration of ARD intake (yr)**	1.25	1.10-1.41	<.001

**Table 4:** Intervention level characteristics (*n* = 185).

**Variable**	**Subgroup**	***N***	**%**	**M**	**SD**
**Gender**	Male	19	10.27		
	Female	166	89.73		
**Tobacco smoking**	Yes	15	8.11		
**Autoimmune disease**	Yes	13	7.03		
**Hypertension**	Yes	79	42.70		
**Hypothyroidism**	Yes	35	18.92		
**Diabetes mellitus**	Yes	59	31.89	6.83	0.97
**Asthma or COPD**	Yes	11	5.95		
**Ischemic heart disease**	Yes	32	17.30		
**Hyperlipidemia**	Yes	75	40.54		
**Bone graft**		10	5.41		
**Implants exposed**	0	157	84.86	0.23	0.64
	1	20	10.81		
	2	4	2.16		
	3	2	1.08		
	4	2	1.08		
**Implants placed**	0	151	81.62	0.59	1.44
	1	10	5.41		
	2	13	7.03		
	3	4	2.16		
	4	1	0.54		
	9	4	2.16		
	11	2	1.08		
**Extractions per treatment**	0	52	28.11	1.54	1.89
	1	70	37.84		
	2	31	16.76		
	3	12	6.49		
	4	11	5.95		
	6-11 (each)	2-2	~1.08		
**Location of treatment**	Mandible	90	48.65		
	Maxilla	95	51.35		
**MRONJ**	Yes	14	7.57		
	No	171	92.43		
**Age (yr)**				72.09	8.34
**Number of injections**				6.13	4.36
**Duration of ARD intake (yr)**				8.18	5.13
**Dentoalveolar intervention delay^a^ (mo)**				5.37	1.49
** MRONJ group** ** Non-MRONJ group**				2.03 5.74	0.75 0.95

a Dentoalveolar intervention delay − interval between last Dmab injection to dentoalveolar intervention.

**Table 5:** Intervention level univariate logistic regression analysis for MRONJ (*n* = 185).

**Variable**	**Subgroup**	**OR**	**95% CI**	***p*-value**	**MRONJ ratio**
**Gender**	Female	1.70	(0.20-14.70)	.6308	7.83%
**Tobacco smoking**	Yes	2.09	(0.35-12.60)	.4219	13.33%
**Autoimmune disease**	Yes	0.94	(0.10-8.69)	.9579	7.69%
**Hypertension**	Yes	0.75	(0.23-2.39)	.6242	6.60%
**Hypothyroidism**	Yes	3.20	(0.96-10.70)	.0592	17.14%
**Diabetes mellitus**	Yes	0.36	(0.07-1.76)	.2059	3.39%
**Asthma or COPD**	Yes	2.51	(0.46-13.83)	.2893	18.18%
**Ischemic heart disease**	Yes	0.35	(0.04-2.94)	.3321	3.13%
**Hyperlipidemia**	Yes	1.63	(0.51-5.27)	.4107	9.33%
**Bone graft**		N/A	N/A	N/A	No MRONJ cases
**Implant exposure**		N/A	N/A	N/A	No MRONJ cases
**No. of implants placed**		0.80	(0.60-1.05)	.1122	–
**Previous presence of implant (yes/no)**	Yes	2.45	(1.02-5.86)	.044	13.51%
**Extractions per treatment**		0.95	(0.80-1.12)	.533	–
**Extractions (yes vs no)**	Yes	3.36	(0.79-14.20)	.100	9.41%
**Location of treatment**	Maxilla	1.77	(0.67-4.70)	.250	9.47%
**Age (yr)**	–	1.07	(1.00-1.14)	.0525	–
**Number of injections**	–	1.10	(0.95-1.27)	.1912	–
**Duration of ARD intake (yr)**	–	1.19	(1.05-1.36)	.0077	–
**Dentoalveolar intervention delay (mo)**	–	0.26	(0.12-0.57)	.0008	–

Following multivariable logistic regression analysis at the intervention level (Table 6), and after assessing for multicollinearity, 2 key factors remained independently and significantly associated with MRONJ development were duration of ARD intake (OR 1.33, 95% CI: 1.07-1.65, *p* = .0101) and dentoalveolar intervention delay (OR 0.35, 95% CI: 0.16-0.77, *p* = .0089).

**Table 6:** Multivariate logistic regression analysis for MRONJ (intervention level).

**Variable**	**OR**	**95% CI**	***p*-value**
**Hypothyroidism**	2.17	(0.19-24.29)	.5286
**Previous presence of implants**	6.30	(0.81-49.15)	.0790
**Age (yr)**	1.13	(0.91-1.40)	.2827
**Duration of ARD intake (yr)**	1.33	(1.07-1.65)	.0101
**Dentoalveolar intervention delay (mo)**	0.35	(0.16-0.77)	.0089
**Extractions (yes vs no)**	2.71	(0.03-238.40)	.6620

No statistically significant association was found between the specific type of dentoalveolar procedure (eg, implant exposure, tooth extraction, bone graft, and implant placement) and the development of MRONJ (*p* > .05).

## Discussion

Through an examination of the incidence, risk factors, and clinical outcomes related to Dmab-induced osteonecrosis of the jaws (DRONJ) in our facility, we aim to guide clinicians in making well-informed management decisions. This approach focuses on achieving a balance between the benefits of osteoporotic fracture prevention and minimizing the risk of MRONJ.

Our retrospective analysis identified 258 osteoporotic patients treated with Dmab within a larger cohort of 1916 osteoporotic individuals. Medication-related osteonecrosis of the jaw was observed in 5.43% of the total cohort, while among the subset of 91 patients who underwent surgical interventions (averaging 2 interventions per patient) involving bone (eg, extractions, implant placements/extractions/exposures, and bone augmentation), and 14 developed MRONJ (15.4%). No cases were observed in patients who did not undergo surgery. This aligns with existing evidence that invasive dental procedures serve as significant precipitating factors for MRONJ development in patients on antiresorptive therapy.^6^^,^^7^^,^^16^

All MRONJ cases in our cohort occurred when surgical interventions took place within 2 mo of Dmab injection, while multivariable analysis (Table 6) specifically highlighted dentoalveolar intervention delay as a critical independent protective factor (OR = 0.35). The OR of 0.35 for indicates a strong protective effect: The odds of developing MRONJ were reduced by 65%, suggesting a temporal relationship where perioperative timing critically influences risk for MRONJ occurrence. This finding corroborates the hypothesis that MRONJ risk peaks during periods of suppressed bone turnover immediately following antiresorptive therapy. This is further underscored by our finding that the number of Dmab injections was not a significant independent predictor of MRONJ occurrence at either the patient or intervention level, emphasizing the crucial role of timing over cumulative exposure of Dmab. The role of cumulative dosage in determining MRONJ risk, while well-established for patients on nitrogen-containing BPs (N-BPs), may not hold true for those receiving Dmab. This is because Dmab’s mechanism of action differs fundamentally from BPs; it does not integrate into the bone matrix by binding to hydroxyapatite. This lack of permanent incorporation allows bone turnover to recover rapidly after the drug is cleared from the system, suggesting that the timing of administration is a more critical factor than the cumulative dose.^22^ While studies such as Park et al. have shown that cumulative BP dose over 1 yr increases MRONJ risk, the same study found no significant difference in risk between patients with 1-4 yr of cumulative BP use vs those with over cumulative 4 yr of use. It is important to note that these findings were based solely on BPs and may not apply to Dmab due to its different mechanism of action.^23^ Another study by Park et al. specifically investigated the timing of dentoalveolar intervention in patients administered i.v. BPs, and found that pausing treatment for over 90 d substantially lowered the risk of MRONJ, with the lowest risk seen after a pause of more than 1 yr. However, this study did not include Dmab due to a lack of available data, and the authors suggested that further research on Dmab specifically was essential.^24^ Campisi et al. proposed in her study a “pragmatic window”—around 5-7 mo after Dmab injection—during which bone remodeling begins to recover, potentially lowering the risk of MRONJ. The European Calcified Tissue Society (ECTS) also suggesting a surgical window at 5-6 mo post-injection when bone turnover effects are most depleted.^18^^,^^25^ However, a significant clinical dilemma arises with these approaches. From an osteoporosis management perspective, this “pragmatic window” is problematic because it necessitates postponing the next Dmab injection, a delay that places the patient at a heightened risk for a rebound-related fracture. This vulnerability is further extended by the need to wait for adequate mucosal healing following the dentoalveolar surgery, intensifying the period of increased fracture susceptibility. The decline of Dmab, follows a biphasic pattern: an initial phase characterized by a linear decrease in serum concentrations from the peak, followed by a more rapid terminal phase with an average serum half-life of approximately 25-30 d.^10^ The reversibility of its pharmacodynamic effects, as indicated by serum bone turnover markers, is observed at the end of the dosing interval. Notably, serum C-terminal telopeptide of type I collagen (CTX) levels decrease by over 80% within 5 d post-dosing and then begin to gradually rise from around 4 mo.^26^ However, the reversible impact of Dmab on BMD supports the concern of an increased fracture risk, known as the “rebound phenomenon,” following discontinuation of the medication in osteoporosis patients. Recent observational studies estimated a vertebral fractures risk 3- to 5-fold higher in patients off-treatment compared to those who continued treatment with Dmab.^27^^,^^28^ The risk of vertebral and multiple vertebral fractures after Dmab discontinuation increases with longer treatment durations, which is consistent with evidence of greater bone loss and a stronger “rebound” in bone turnover following extended use or a higher number of doses.^29^ This effect generally occurs between 8 and 16 mo after the final Dmab injection and should be carefully considered when planning dentoalveolar surgical procedures.^19^^,^^22^ Our multivariable model found no significant association between the specific type of dentoalveolar procedure (eg, tooth extraction, implant placement, bone grafting, or implant exposure) and MRONJ risk. This observation reinforces the central finding that the perioperative timing of Dmab administration is the primary determinant of MRONJ risk, irrespective of the specific dentoalveolar procedure. This finding contrasts with BPs, for which a considerable body of evidence suggests that cumulative dose is a key risk factor.^23^ Interestingly, our data indicated a higher risk associated with dental implants (OR 2.45). It is critical to clarify that this risk was primarily associated with the development of MRONJ at the sites of existing, previously integrated dental implants rather than being limited to the surgical placement phase. This finding aligns with the systematic review by Nisi et al., which identifies dental implants as a distinct risk factor for MRONJ, often arising from chronic peri-implantitis an average of 46.5 mo after placement.^30^ Furthermore, the recent systematic review and consensus statement by Ali et al., reported that while 38% of implant-related MRONJ cases occur during the initial surgical phase, the majority (62%) develop after the implants have been loaded and are functional.^31^ This distinction is vital when interpreting this systematic review finding that antiresorptive therapy shows no statistically significant association with “implant failure” (loss of osseointegration). Our data suggests that while implants can successfully integrate and remain functional, they concurrently serve as a local focus for bone necrosis due to chronic inflammatory triggers. A key factor in this susceptibility may be the unique anatomical and physiological profile of peri-implant tissues. Unlike natural teeth, which are buffered by a periodontal ligament (PDL) that provides a robust vascular supply and an organized immune response, dental implants lack a PDL.^32^ This structural difference results in a more direct interface between the oral environment and the bone, potentially making the peri-implant site more vulnerable to chronic inflammatory triggers.^33^ This emerging entity, termed peri-implant MRONJ (PI-MRONJ), represents a rising clinical challenge that warrants further research to elucidate its specific pathophysiology, necessitating both meticulous surgical timing for new placements and rigorous, lifelong monitoring of existing fixtures.

Contrary to the prevailing literature, most MRONJ cases in our study localized to the maxilla (9 cases) rather than the mandible (5 cases) (see Supplementary Material S1).^6^^,^^7^^,^^34^ This unexpected finding may relate to the distinct vascular anatomy of the maxilla, which is richer in vasculature, and the proposed mechanism involving Dmab, which does not penetrate or bind directly to the bone mineral as BPs.^35^^,^^36^ Instead, it originates from and is transported through the vasculature, which is more abundant in the maxilla. Another possible explanation for our findings is the role of the inflammatory process in MRONJ. The primary goal of inflammation is to repair damaged tissue and restore cellular homeostasis.^37^ During this process, leukocytes release pro-inflammatory cytokines, such as VEGF, IL-1α, IL-1β, and TNF-α, which, along with chemokines, target endothelial cells.^38^ This interaction can induce vasodilation and increase vascular permeability.^39^ Given the maxilla’s more abundant vasculature, this inflammatory response may be amplified, acting as a co-factor in MRONJ development, as suggested by previous studies.^10^ Further research is needed to fully elucidate this hypothesis.

Our data also suggest that most MRONJ cases presented at stages 1 or 2 (13 cases), with only a single stage 3 case (see Supplementary Material S1), potentially reflecting the mitigated severity due to the pharmacokinetics of Dmab. Since it does not bind directly to bone mineral and serum levels decline within approximately 4 mo after the Cmax (the maximum concentration of the drug in the blood plasma), the extent of osteonecrosis may be limited, leading to better prognosis.^40–42^ This is further supported by studies that have distinguished histopathological and radiologic features, as well as clinical outcomes, between DRONJ and BP-related osteonecrosis of the jaw (BRONJ). DRONJ has been shown to have significantly fewer osteocytes per unit area,^43^ whereas persistent bone resorption lacunas on the necrotic bone surface were observed exclusively in BRONJ cases.^44^ Radiologically, DRONJ may present differently from BRONJ, often showing fewer sequestra and less cortical lysis.^45^ Additionally, it has been proposed that DRONJ may heal more rapidly, potentially due to its shorter duration of treatment effect and reversible inhibition of RANKL, which could influence its overall healing dynamics.^46–48^ It is essential to distinguish between the risk profiles of oncology and osteoporosis cohorts. In oncology patients receiving high-dose (120 mg), monthly Dmab, advanced mandibular involvement is frequently reported due to the profound and continuous suppression of bone turnover, due to higher cumulative dose administered.^25^ In contrast, our cohort received a much lower (60 mg) dose with a 6-mo dosing interval. Despite the mandible’s limited vascularity, we hypothesize that the significantly higher cumulative doses administered in oncology protocols successfully penetrate the mandibular bone, which is inherently more prone to MRONJ, thereby inducing profound and sustained turnover suppression.

Our observation that most patients developing MRONJ had a history of prolonged (average 14.58 yr) treatment with ARDs including previous treatment with BPs, suggests a synergistic or cumulative risk, supported by meta-analyses indicating higher MRONJ prevalence in patients transitioning from BPs to Dmab.^49^ All 14 patients who developed MRONJ had prior prolonged BP exposure (100%). This finding is crucial and a significant confounding factor, strongly suggesting that the risk profile observed in our study is not solely due to Dmab, but rather a reflection of the cumulative risk associated with sequential antiresorptive therapy. The long-term retention of BPs in the bone matrix, combined with the potent, though reversible, suppression of turnover by Dmab, likely creates a highly susceptible state for MRONJ development following surgery. Our analysis identified MRONJ in 5.43% of the total cohort, a frequency that is notably higher than often cited. However, this finding is consistent with recent “real-world” data reported by Everts-Graber et al., who observed a significantly higher incidence of MRONJ in patients transitioning from BPs to Dmab compared to those receiving Dmab alone.^16^While Everts-Graber et al. noted an increased risk in patients with a mean previous BP duration of 6.7 yr, our cohort exhibited an even more extensive history of pretreatment, with an average of over 9 yr of BP therapy prior to Dmab administration. This underscores a synergistic risk profile: The long-term skeletal retention of BPs combined with the bone turnover suppression of Dmab creates a highly susceptible state for necrosis following dentoalveolar surgery. Furthermore, our status as a tertiary medical center likely contributes to this higher percentage, as our facility frequently manages complex cases and patients with significant comorbidities who are referred after developing complications from dental treatments performed elsewhere. The prolonged suppression of bone remodeling predisposes to necrosis following surgical procedures involving bone, underscoring the importance of comprehensive medication history and rigorous risk assessment prior to surgical intervention. We did not evaluate the influence of the specific chemical subclass of BP, as the vast majority of our pretreated patients received oral BPs. The specific pharmacokinetic influence of different BP molecules on subsequent Dmab treatment remains a subject for future dedicated study.

**Figure 2:**
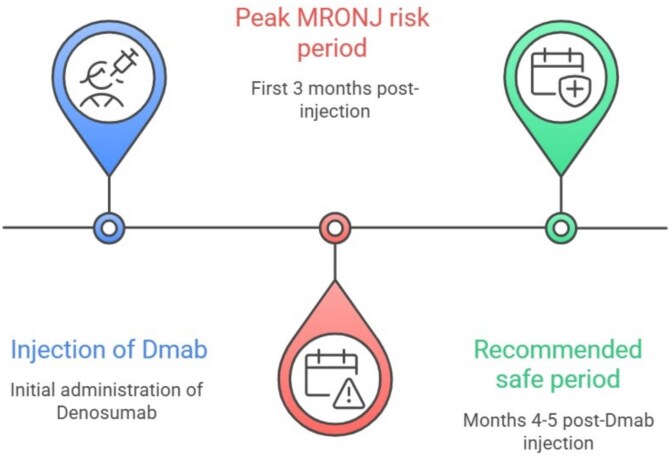
Timeline for recommended “safe period” for dentoalveolar intervention in patients receiving Dmab treatment.

Overall, these findings highlight the complex interplay between treatment timing, prior medication history, and anatomical factors in MRONJ development. They underscore the need for careful planning of surgical interventions, ideally within the identified “window” of bone healing post-Dmab, to mitigate risks. The latest Italian SIPMO-SICMF position paper on MRONJ recommends that dentoalveolar surgery be performed approximately 5 mo after the last Dmab injection, with a 1-mo delay before administering the next injection, until complete healing is confirmed. A recommendation based on expert opinion rather than clinical data.^7^ In our study, and consistent with the pharmacological data presented, no cases of MRONJ occurred when interventions were carried out at least 4 mo after the most recent Dmab injection. Based on our clinical experience, mucosal healing can take 6-8 wk, especially after more complex procedures. Therefore, we advise against elective bone-related surgeries within the first four months following Dmab treatment (see Figure 2). Our findings, based on clinical extrapolation, suggest that the optimal timing for surgery is between 4 and 5 mo post-injection. While the ECTS guidelines^25^ recommend a window of 5-6 mo, we propose that a 4-mo threshold offers a superior clinical balance. Performing surgery at the 5-to-6-mo mark, as suggested by the ECTS, inevitably pushes the subsequent Dmab dose to month 8 or 9 due to the necessary 6-8 wk healing period. This delay significantly extends the patient’s exposure to the “rebound effect.” Further prospective studies are essential to validate these observations and refine clinical guidelines for optimal timing of surgical procedures in osteoporotic patients on Dmab therapy.

This study benefits from a well-characterized cohort of osteoporotic patients treated with Dmab, including detailed data on surgical interventions and MRONJ outcomes. A key strength of our study is the diverse patient population, with a broad range of comorbidities and a history of long-term exposure to antiresorptive agents, including BPs. This provides valuable insights into MRONJ risk in a complex, real-world patient population. However, the retrospective design has inherent limitations, including reliance on medical records for data extraction from a single medical center, and the potential for associated reporting or documentation bias. Additionally, the relatively small number of MRONJ cases limits the statistical power for identifying independent risk factors in multivariable analyses.

### Conclusions

This retrospective study confirms that surgical timing is the critical determinant of MRONJ risk in osteoporotic patients taking Dmab. All MRONJ cases occurred when dentoalveolar interventions were performed within 3 mo of a Dmab injection, while an increased interval between the injection and dentoalveolar intervention, was identified as a significant protective factor. Prolonged antiresorptive drug intake was found to be a possible contributor to MRONJ development. We conclude that delaying dentoalveolar procedures for at least four months post-injection, especially after previous long term ARD intake, appears to be a crucial preventive measure, as it minimizes the risk of MRONJ without triggering the fracture risk associated with prolonged drug discontinuation.

## Supplementary Material

Supplementary_material_MRONJ_cases_related_characteristics_ziag038

## Data Availability

The data underlying this article were accessed from “Beilinson Hospital” medical records. The data underlying this article cannot be shared publicly due the privacy of individuals that participated in the study. The data will be shared on reasonable request to the corresponding author.
