# Supplementary material for: The therapeutic window: timing dentoalveolar surgery to minimize MRONJ risk in denosumab-treated osteoporotic patients
Source: JBMR Plus. 2026 Mar 12;10(5):ziag038. doi: 10.1093/jbmrpl/ziag038 (PMC13050504; doi:10.1093/jbmrpl/ziag038)
Supplement: Supplementary_material_MRONJ_cases_related_characteristics_ziag038 [file supplementary_material_mronj_cases_related_characteristics_ziag038.docx]

*MRONJ cases related characteristics (n=14)*

| **Patient** | **Gender** | **Age (at osteoporosis diagnosis)** | **Timing of DA intervention after last Dmab injection (months)** | **Number of Dmab injections** | **Duration of ARD intake (years)** | **Jaw of MRONJ development** | **Stage of MRONJ** |
| --- | --- | --- | --- | --- | --- | --- | --- |
| 1 | Female | 76 | 2 | 6 | 23 | Mandible | 2 |
| 2 | Female | 73 | 3 | 12 | 6 | Maxilla | 1 |
| 3 | Female | 77 | 2 | 14 | 21 | Maxilla | 1 |
| 4 | Female | 62 | 2 | 7 | 13 | Maxilla | 2 |
| 5 | Female | 83 | 2 | 5 | 4 | Maxilla | 1 |
| 6 | Female | 84 | 3 | 18 | 19 | Maxilla | 1 |
| 7 | Female | 80 | 3 | 4 | 4 | Maxilla | 2 |
| 8 | Female | 87 | 1.5 | 3 | 5 | Maxilla | 2 |
| 9 | Female | 67 | 1 | 6 | 18 | Mandible | 1 |
| 10 | Female | 74 | 1 | 9 | 5 | Mandible | 1 |
| 11 | Male | 84 | 2 | 20 | 17 | Maxilla | 2 |
| 12 | Female | 66 | 2 | 19 | 19 | Maxilla | 2 |
| 13 | Female | 74 | 1 | 6 | 7 | Mandible | 3 |
| 14 | Female | 82 | 3 | 2 | 22 | Mandible | 1 |
